# Functional Architecture of the Human Insula Revealed by Causal Intracranial Mapping

**DOI:** 10.21203/rs.3.rs-8912902/v1

**Published:** 2026-03-08

**Authors:** Josef Parvizi, Julian Quabs, Sofia Pantis, Guanpeng Chen, Weichen Huang, Eileen Ma, MARIA DEL VECCHIO, Dian Lyu, Chloe Wang, Pietro Avanzini, Vivek Buch, Ashwin Ramayya, Svenja Caspers, Hannes Vogel

**Affiliations:** Stanford University; Stanford University; Stanford University; Stanford University; Stanford University; Stanford University; Istituto di Neuroscienze, Consiglio Nazionale delle Ricerche; Stanford University; Stanford University; Istituto di Neuroscienze, Consiglio Nazionale delle Ricerche; Stanford University; Stanford University; Research Centre Jülich; Stanford University Medical Center

**Keywords:** limbic, electrical stimulation, cytoarchitecture, sEEG, electrocorticography, ECoG, intracranial EEG, subjective experience, connectivity, cerebrocerebral evoked potentials, CCEP, repeated single pulse, insula

## Abstract

The insular cortex plays a central role in pain, emotion, and cognition, yet its functional architecture and causal electrophysiological relationships among its subregions remain poorly understood. Here, we integrated intracranial electrical stimulation and task-based electroencephalography recordings with connectivity mapping within a cytoarchitectonic atlas of the human insula in 87 neurosurgical participants, identifying a quadripartite functional architecture comprising functionally distinct regions: (i) dorsal-posterior region encoding somatotopically organized nociceptive/thermoceptive and somatosensory signals; (ii) ventral-posterior region integrating somatic information across multiple body parts; (iii) mid-anterior region associated with visceral sensations and anxiety states; and (iv) anterior-polar region, largely silent to direct stimulation, yet showing robust activation during salience detection and change of action mode. Critically, this anterior region, with its strong connections with the prefrontal cortex including anterior cingulate cortex, exerts strong and direct influence over other insular regions, while receiving less strong indirect inputs from them, revealing an electrophysiological pathway for cognitive modulation of pain and bodily perception. Together, these findings define a functional architecture of the human insula that links cytoarchitecture to directed and asymmetric electrophysiological interactions with mechanistic implications for cognitive modulation of pain and interoceptive experience.

## INTRODUCTION

Interoception refers to the brain’s ability to sense, interpret and integrate bodily signals such as pain, a function that is essential for survival and well-being. Interoception was first defined by Sherrington over a century ago as the sensing of internal bodily states, distinct from exteroception and proprioception^1^. Subsequent work established the insular cortex as a primary hub for interoceptive processing and for linking bodily signals with multiple large-scale brain systems^2–4^. Evidence from anatomical tracing studies in non-human mammals showed that the spinal pain and temperature pathways project preferentially to the dorsal fundus of the posterior insula rather than to primary somatosensory cortex^5^. With the advent of neuroimaging, a model was proposed that posited a “posterior-to-anterior flow of information” originating from primary interoceptive signals in the posterior insula to integrated bodily representations and subjective awareness in the anterior insula^6^. However, this framework has been challenged by lack of causal experimental evidence while anecdotal lesion evidence has documented preserved subjective feelings despite extensive bilateral anterior insular damage^7^. To date, it remains unclear how interoceptive signals are organized and transformed within the human insula, and which subregions directly access subjective conscious states.

While the prevailing model of insular functions, based on the notion of interoception, emphasizes a largely bottom-up (i.e. posterior to anterior) flow of information processing, an alternative framework of “interoceptive inference”^8^ proposes an active ‘top-down’ processing within the framework of predictive coding^9,10^. In this framework the insular cortex, with strong top down inputs from the prefrontal cortices, supports error-based learning of feeling states and uncertainty^11^ and as responding to interoceptive mismatches that underlie anxiety^12^. To date a causal examination of these two seemingly opposite views has remained to be determined.

Prior causal studies of the insula with the lesion method have indicated a dual functional architecture on opposite sides of the central sulcus of the insula: anterior insular damage has been commonly associated with affective, motivational, and language-related disturbances while posterior insular lesions have preferentially been associated with problems with pain, temperature, and sensory processing^13,14^. Another line of causal evidence about insular functions relates to intracranial electrical stimulation of the insula in neurosurgical participants^15–30^. These studies reported somatosensory, thermoceptive, and nociceptive responses across the posterior insula; visceral chemosensory effects, such as taste and olfactory sensations near and in the depth of the central sulcus; and throat/ and gastrointestinal sensations, tachycardia and nausea in the anterior insula. Only anecdotes of fear and anxiety^27,30^ or ecstatic^31^ feelings during insular electrical stimulation have been reported.

Previous studies, while offering important insights, have been limited in their approach: Most prior studies have examined the insula through isolated anatomical, functional, or connectivity lenses. Here, we unify these dimensions using a high-resolution and multimodal approach that enables an unprecedented characterization of its functional architecture using electrophysiological causal measures of its function and connectivity with not only the broader cortical mantle but also among its different subregions.

Traditionally, the insula has been divided to anterior versus posterior divisions, or at the level of its gyri. Contrarily, microstructural studies have divided the insula to granular, dysgranular, and agranular subregions going beyond the simple anterior-posterior subdivisions^32,33^. However, it is important to note that the previous anatomical maps were primarily 2D drawings based on limited sample sizes^34^ that are incompatible with modern 3D reference space. Addressing these limitations, a recently developed cytoarchitectonic atlas has provided novel quantitative insights into insular microstructural architecture^4,35–38^. This parcellation has revealed 16 distinct cytoarchitectonic areas exhibiting microstructural diversity that transcends traditional layer IV-based classification beyond the macroscopic anatomical boundaries.

In the present study, we explore the functional architecture of the insula with a multimodal approach using this newly developed microstructural atlas of the human insula. In a cohort of 87 human participants implanted with intracranial electrodes, our current work aims to understand the fundamental organizing functional principles of insular cortex in the human brain in light of the new evidence about its microscopical anatomical architecture. Our observations reveal a striking correspondence between microstructural architecture and function, providing rare causal alignment among cytoarchitectonic regions, stimulation-evoked subjective phenomenology, direct recordings of local field potentials during experimental settings, and directional connectivity.

## RESULTS

### Participants and Anatomical Coverage

This study includes 87 participants who underwent stereoelectroencephalography (SEEG) electrode implantation with intracranial electrodes (AdTech) for seizure localization at Stanford University Medical Center between 2012 and 2025: 53 male, 34 female, aged 19–67 years (38 ± 12.2 years).

Functional mapping of the insula with intracranial electrical stimulation electroencephalography - using high frequency (50Hz) pulses - was performed in 50 participants. These participants were implanted with 478 electrodes (9.56 ± 7.07 per subject), a relatively high number reflecting preferential use of oblique trajectories through the insular cortex^39^. Only ten participants had a seizure onset zone localized to the insula; in these participants we excluded all epileptogenic channels from subsequent functional and connectivity analyses. The remaining electrodes were balanced across the hemispheres (234 left, 244 right) and anterior-posterior (237 anterior, 241 posterior) subdivisions. Individual participants’ electrode coverage was either left-only (19 participants), right-only (10), or bilateral (21); among bilateral cases, hemispheric asymmetry averaged 3.38 ± 3.97 electrodes. Anterior-posterior coverage could be categorized as anterior-only (13 participants), posterior-only (7), or spanning both subdivisions (30); subdivision asymmetry averaged 4.13 ± 4.30 electrodes. Collectively, these distributions demonstrate balanced sampling across both hemispheres and anatomical subdivisions of the insular cortex.

In the stimulation experiments, we only included participants’ subjective reports if they passed our strict quality control metrics: Reports were (1) evokedonly during real, but not sham, stimulations, (2) reproducible when the same site was repeatedly stimulated, and (3) dose–dependent (i.e., effect intensity increased as stimulation intensity increased).

### Divergent Responses to Intracranial Electrical Stimulation Across Insular Regions

**Figure 1:**
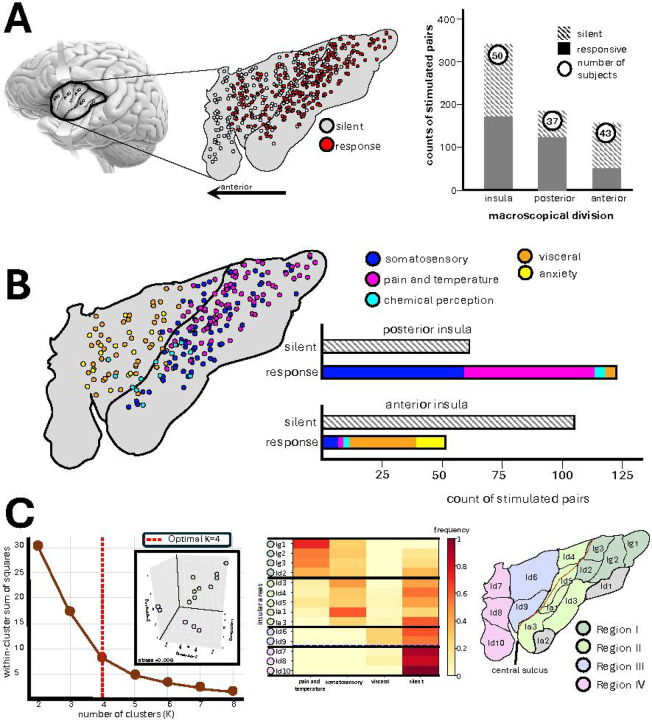
Distinct Subjective Responses Within the Human Insula. **A)**Electrode coverage of the human insula displayed on flat map template with sites color-coded as responsive (red) or silent (gray) to electrical stimulation. The bar plot shows counts of responsive and unresponsive electrodes and number of participants for each division, revealing higher overall responsiveness in the posterior insula (67%) compared to the anterior insula (32%). **B)** Categorization and spatial distribution of responsive electrodes are shown across five distinct categories of subjective states induced by electrical stimulation: somatosensory, pain and temperature, chemosensory perception, visceral, and anxiety. Posterior insula stimulation predominantly evoked somatosensory, pain and temperature whereas anterior insula stimulation exclusively elicited visceral and anxiety-related responses. Chemosensory sensations were induced by the stimulation of the cortices deeper in the ventral central sulcus. **C)** Given the fine-grained parcellation of the insula into 16 cytoarchitectonic areas, we investigated how these microstructural regions organize functionally based on stimulation-evoked responses. Electrode contacts were registered to a cytoarchitectonic atlas of 16 insular areas (grey areas, Id1 and Ia3, indicate no electrode coverage). Response frequencies were calculated for each area across functional categories (somatosensory, pain/temperature, visceral, no response), including only features with >5% overall elicitation rate. Unsupervised k-means clustering identified four regions (optimal cluster number determined by elbow method; validated by 3D multidimensional scaling, stress=0.01). Color intensity in the heat map reflects response frequency per area for each category. Region I (Ig1, Ig2, Ig3, Id2) exhibited predominant pain and temperature responses. Region II (Id3, Id4, Id5, Ia1, Ia2) showed primarily somatosensory responses. Region III (Id6, Id7) demonstrated highest visceral response frequencies. Region IV (Id8, Id9, Id10) was silent to electrical stimulation. These data reveal a four-fold functional organization of the human insula based on evoked electrical response profiles of microstructural areas.

First, we compared the effects of electrical stimulation of the traditional anterior vs. posterior insular organization across 342 pairs of adjacent electrodes in 50 participants, of which 159 pairs (43 participants) were located in the anterior, and 184 (37 participants) were located in the posterior division of the insula. The anatomical division of anterior vs posterior insula was made in native anatomical space using the central sulcus as the landmark of interest. Subjective responses were evoked by electrical stimulation in 51% of insular sites, with a higher elicitation rate in the posterior (67%) compared to the anterior insula (32%)(χ^2^ test, p<0.0001, OR=4.23, 95% CI [3.07–5.82]) (Figure 1).

All Subjective reports induced by electrical stimulation of the insula could be grouped into five clear categories: somatosensory (i.e., tingling, numbness, or pressure sensations, 67 pairs, 16 participants), pain and/or temperature (i.e., noxious or heat sensations occurring either separately or together, 56 pairs, 15 participants), chemical perception (i.e., taste and olfaction, 7 pairs, 6 participants), visceral (i.e., internal bodily sensations of nausea, dizziness, wave-like energy in the body core, throat or head, 33 pairs, 14 participants), and emotional (mostly anxiety and nervousness, 11 pairs, 5 participants) with some participants contributing to more than one category of responses. Somatosensory, pain and temperature and chemical sensations were predominantly associated with the electrical stimulation of the posterior insula while visceral and anxiety responses were exclusively localized to the anterior insula (stats shown in Figure 1).

We explored the lateralization of the induced effects by analyzing data from only participants who had bilateral insular implementations. Our data suggested that the effects from anterior (13 participants, 30.6% left, 31% right, p=0.76) or posterior (11 participants, 74.1% left, 87.4% right, p=0.06) sites were not significantly different. One exception was the finding of anxiety responses from the stimulation of left (but not right) anterior insula. However, only three of the five participants had bilateral electrodes and the lateralization of anxiety responses to the right hemisphere needs future validation.

Given that a recently developed cytoarchitectonic map revealed a more fine-grained parcellation of the insula into 16 distinct areas, we examined how these microstructural areas group together based on their electrically evoked response profiles to potentially form functionally similar units. First, we registered the cytoarchitectonic atlas to each subject’s T1-weighted image in native space, labeled each electrode contact according to its cytoarchitectonic area, and second, calculated response frequencies across functional categories (somatosensory, pain/temperature, visceral, no response). Analysis was restricted to features with >5% overall elicitation rate to ensure reliability.

Unsupervised k-means clustering identified four functional/structural regions (Figure 1C) (optimal cluster number determined by elbow method; validated by 3D multidimensional scaling, stress=0.01). Region I (dorsal-posterior insula: Ig1, Ig2, Ig3, Id2) exhibited predominantly pain and temperature responses. Region II (ventral-posterior insula: Id3, Id4, Id5, Ia1, Ia2) showed primarily somatosensory responses. Region III (mid-anterior insula: Id6, Id7) demonstrated the highest frequency of visceral response and Region IV (anterior pole: Id8, Id9, Id10) was largely unresponsive to direct electrical stimulation. These findings reveal a four-fold structural-functional organization of the human insula based on electrically evoked response profiles of microstructural areas.

### Pain, Temperature, and Somatosensory Responses in Posterior Insular Regions I and II

Stimulation of the posterior and dorsal insula reliably elicited pain and thermosensory percepts – as well as somatosensory changes as detailed above. However, we observed an intriguing meso-scale functional division among anatomically adjacent neuronal populations with distinct yet related roles: while stimulation of some sites evoked pain alone, stimulation of other sites within the same region produced purely thermosensory sensations, stimulation of a third set of sites elicited both warmth and pain, and stimulation of yet another set of sites elicited somatosensory sensations in the absence of pain or temperature percepts. It should be noted that the average electrical current eliciting pain and temperature was comparable with all other responses (pain and temperature: median 3 mA, IQR 2–4mA/all other responses: median 4 mA, IQR 2–6) (Supplementary Figure 1). Stimulation at no site elicited a sensation of cold.

**Figure 2:**
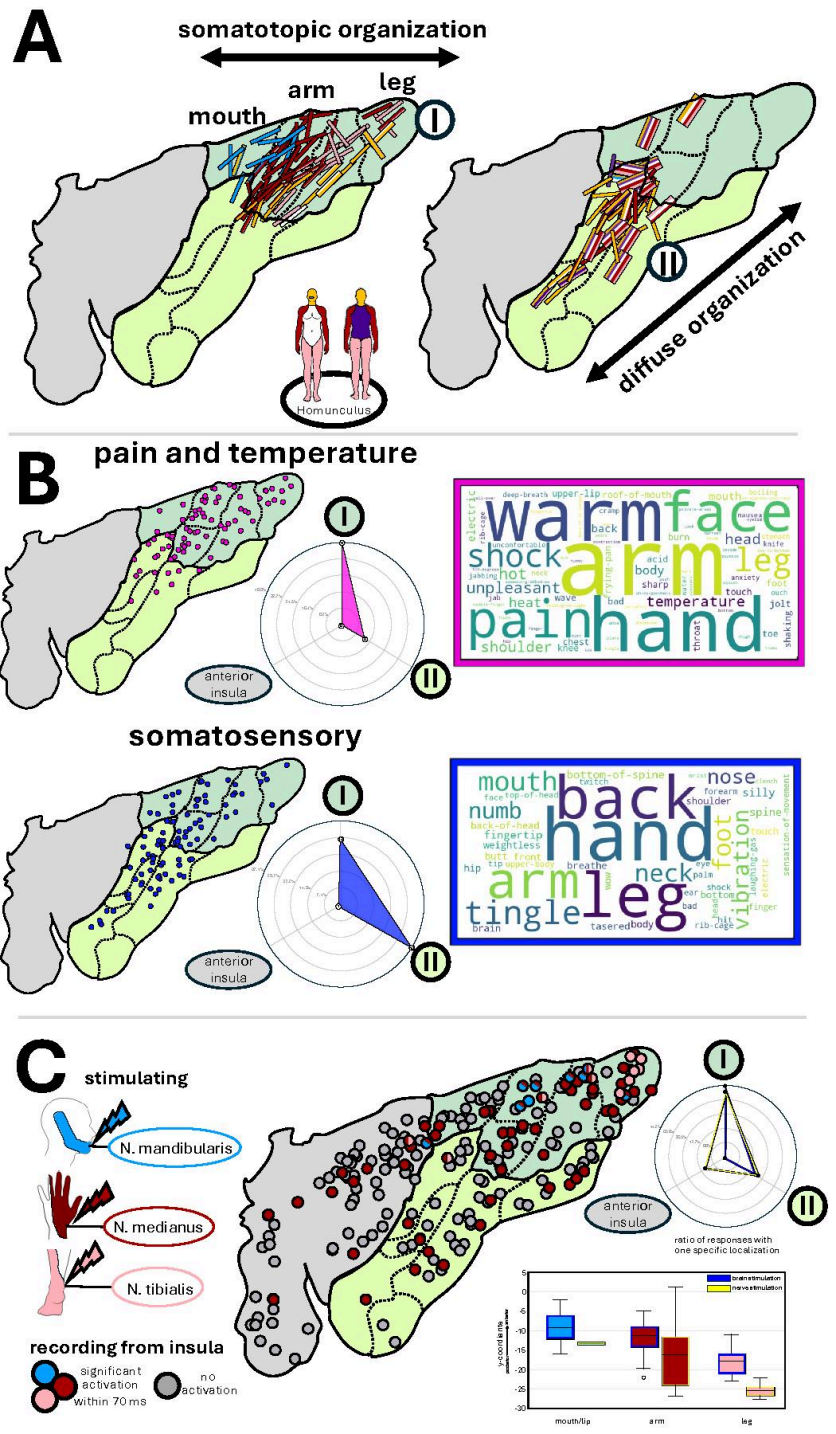
The Posterior Insular Regions I and II and Pain, Temperature, and Somatosensory Sensations. A) Related body parts of pain, temperature, and somatosensory responses mapped onto a microstructural atlas of the insula. Each line shows the location of electrical stimulations applied between two adjacent electrode contacts. Body parts are color-coded according to the depicted homunculus. Responses within the dorsal posterior insula showed a clear somatotopic organization. In contrast, electrodes in the ventral posterior insula evoked sensations spanning multiple body parts without apparent somatotopic organization. It should be noted that the location of the mouth region (light blue lines) corresponds to parcel Id4 in the Julich atlas as it is microstructurally a transitional area of its own, and different from the most granular dorsal posterior insula. Interestingly we caused somatosensory responses only in oral cavity when this region was stimulated. B) Spatial distribution of somatosensory and pain, thermal responsive electrodes, with word clouds representing the most frequently reported experiences for each sensation type. Radar charts show comparable proportions of somatosensory responses in region I (28.37% of all electrodes) and region II (37.11% of all electrodes). However, pain and temperature responses were substantially more prevalent in region I (40.93% of all electrodes) than region II (12.37% of all electrodes). C) Somatosensory Evoked Potentials (SEPs) recorded from the stimulation of peripheral (mandibular, median, and tibial) nerves. Shown are significant electrode responses within 70 ms post-stimulus. Median nerve (hand) stimulation induced widespread activity across the insula, whereas tibial nerve (leg) and mandibular nerve (mouth) responses localized exclusively to region I. The polar plot shows that, similar to cortical stimulation, the highest proportion of significant responses was found in region I (~40%) compared to region II (~20%). Analysis of anterior-posterior coordinates of peripheral nerve responses confirmed the cortical stimulation somatotopy: legs posterior, upper extremity intermediate, and mouth region anterior.

Importantly, our results revealed a clear somatotopic organization in the posterior dorsal (but not ventral) insula corresponding to Region I – within the boundary of a cluster of smaller cytoarchitectonic areas containing a granular layer IV (i.e., granular cortex with a dense layer IV, which is typical for primary sensory cortices) (Figure 2A).

In Region I, 64% of the pain, temperature or somatosensory responsive sites elicited specific sensations in a *single* body part. The head and ventral trunk were represented in the inferior portion of the dorsal posterior insula. Notably, among these single-body part responses, the arm was the most frequently represented (45.3%), compared to the leg (22.1%), head (12.6%), and mouth (18.9%). Furthermore, 62% of arm responses specifically involved the hand, indicating a disproportionate representation of the hand compared to other body parts. In this region legs were represented posterior towards the parietal opercular edge followed by the upper extremity, while the stimulation of the directly adjacent area Id4 anteriorly evoked sensations within the oral cavity, expanding the somatotopic map above the central sulcus and continuing to the anterior sites (which continues to another region of the insula that is involved in visceral and GI related sensations – see below, and Figure 2 for the location of smaller cytoarchitectonic areas). Notably, we reassigned area Id4 from Region II to Region I despite predominantly somatosensory (rather than pain and temperature) responses. This reclassification was based on i) consistent evoked sensations localized to a single body part (the mouth), and ii) matching the somatotopic organization of Region I, a feature not captured in our initial clustering analysis.

Stimulation of sites ventral to Region I, posterior to and within, central sulcus of insula, corresponding to the dysgranular ventral posterior insula, were associated with sensations spanning three or more body parts (21.9% vs. 5.4% in region I) without a clear somatotopic pattern, suggesting that these sites encode larger body segments.

Among somatosensory responses to stimulation, 53.7% were recorded in Region II, compared with 45.5% in Region I. In contrast, pain- or temperature-related sensations were predominantly localized to Region I (78.5%), with only 21.5% arising from Region II (Figure 2B). Four examples illustrating the sharp transition in conscious experience from somatosensory to pain or temperature across electrodes traversing the border between regions I and II in the single participant brain are shown in Supplementary Figure 2. Importantly, the average electrical current eliciting pain and temperature was comparable between regions (Region I: median 3.0 mA, IQR 2–4 mA; Region II: median 2.6 mA, IQR 2–4 mA) (Supplement Figure 1), indicating that this functional distinction reflects genuine organizational differences rather than differential thresholds.

Across regions I and II, 55.7% of responses included lateralized specifications such as ‘left hand’ or ‘right foot’ (left: 26.4%; right: 26.0%; bilateral: 3.2%),while 44.3% did not specify laterality and only reported responses such as ‘warm feeling in the hand’. Among the cases with specified left or right lateralization, 99.2% involved stimulation of the contralateral insula.

To validate our finding of somatotopic organization in Region I, we analyzed sensory evoked potential (SEP) data that were already collected and partially published^40^. Using this available dataset, we mapped the location of early sensory evoked responses (<70ms) across the insular electrodes implanted in the Italian participants, following stimulation of the contralateral mandibular (face), median (arm), and tibial (leg) nerves in 218 recording sites (130 left, 88 right; 144 posterior, 74 anterior) across 37 participants (Italian Cohort) (Figure 2C). Early SEPs were recorded in the dorsal posterior insula (51.1% of all electrodes), compared to ventral posterior (23%) and anterior insula (15%). Direct comparison of the y-coordinates (posterior-to-anterior axis in the MNI space) for all specific responses in both participant cohorts (IES and SEPs) confirmed that the leg was represented in the most posterior part of the dorsal posterior insula (mean y coordinate: −25.51 ± 1.90 (nerve stimulation)/ −17.90 ± 3.40 (brain stimulation)), followed by the upper extremity (mean y coordinate: −16.47 ± 7.77 (nerve stimulation)/ −11.88 ± 4.33 (brain stimulation)), and the mouth/mandibular region in the most anterior part (mean y coordinate: −13.32 (nerve stimulation)/ −9.17 ± 4.34 (brain stimulation)). Of note, majority of SEP sites in Region I were from median nerve stimulation (69%), compared to 15.5% for both tibial and trigeminal nerves, confirming the intracranial electrical stimulation finding of larger hand representation in region I.

In conclusion, our data confirmed the presence of a somatotopically organized nociceptive/thermoceptive and somatosensory primary interoceptive area in the dorsal posterior (Region I) with a secondary area located more ventrally towards the dysgranular posterior insula (Region II), which we postulate to be a possible site for integration of information across different body parts (Figure 2).

### Visceral and Anxiety Responses in the Mid-anterior Insula

**Figure 3:**
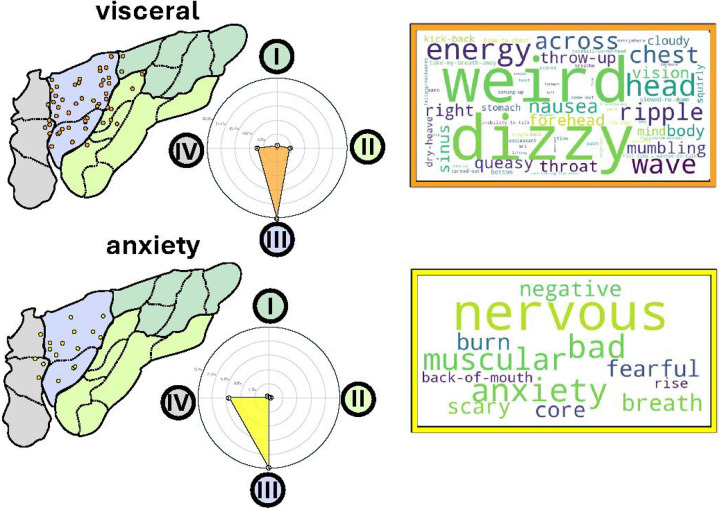
The Mid Anterior Region III and Visceral and Anxiety-Related Sensations. Spatial distribution of visceral and anxiety responsive electrodes, with word clouds representing the most frequently reported experiences for each sensation type. Radar charts show proportions of responsive electrodes for anxiety and visceral responses across regions, with the highest proportions for both functions in region III (visceral 26.8%, anxiety 9.4%).

Stimulation of the anterior insula exhibited a distinct response profile. When mapped onto the microstructural atlas, the majority of responsive sites were localized to the mid-anterior insula, corresponding to Region III of our cluster analysis and encompassing the well-established cytoarchitectonic areas Id6 and Id9 (Figure 3A). Notably, responses evoked by stimulation throughout this region were overwhelmingly confined to the visceral domain. Across 13 participants, 26.8% of electrodes in Region III evoked visceral sensations, in contrast to 0.9% (1 participant) and 4.6% (5 participants) in posterior regions I and II, respectively. Feeling of anxiety was elicited in 5 participants by stimulation of 9.5% of electrodes in region III, compared to 0% in Region I or II, even in those participants experiencing clearly unpleasant pain feelings during stimulation. Participants described negatively valenced responses as rising nervousness and anxiety, whereas visceral sensations were reported as “waves of energy” or motion in the abdominal, thoracic or throat area. The anxiety group showed higher mean Beck Anxiety Inventory scores compared to other response types in the mid-anterior region III (Anxiety: 13.6, Visceral: 7.88, No Response: 11.73; Supplementary Figure 3).

As Region III encompasses dysgranular areas Id6 and Id9 with dense, bipartite output layer V (Va/Vb), and sparse number of cells in the input layer IV, it is reasonable to expect that this region serves as a hub for corticofugal visceral efferents to the thalamus and beyond rather than a receiving sensory region.

### The Silent Anterior Polar Insula

**Figure 4:**
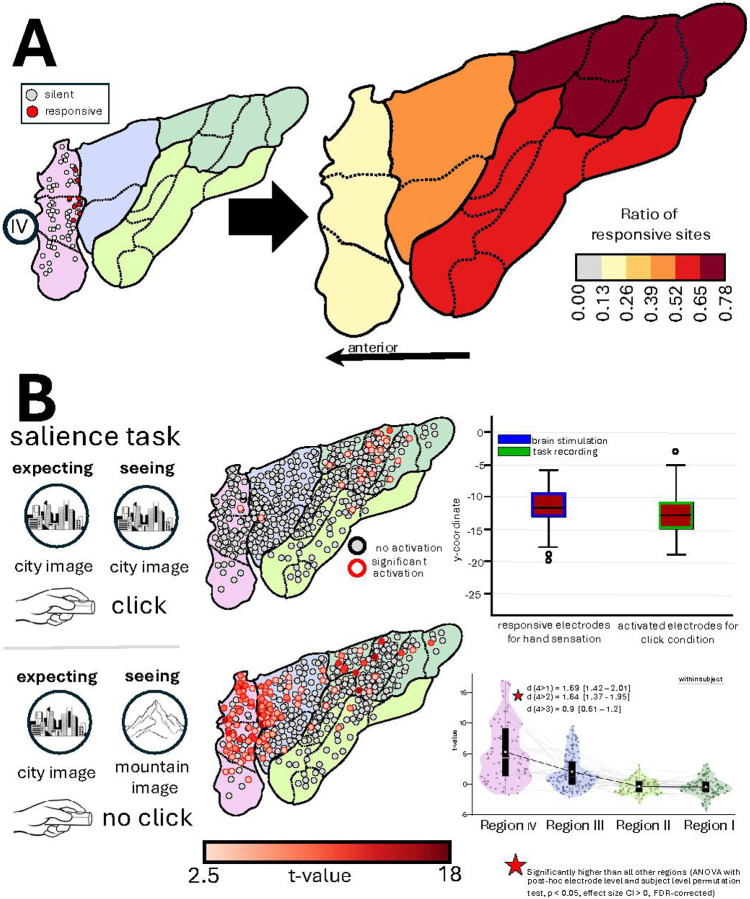
Anterior Polar Region is Silent to Stimulation but Engaged during Salience Detection and Change of Action Mode. **A)** The ratio of responsive sites across each region is color-coded from gray (none) to dark red (very high). Stimulation of 87% of electrodes in Region IV did not result in any reportable change in the subject’s conscious state. By contrast, Region I in the dorsal posterior insula was the most responsive. **B)** Mapping the sites of activation during a Gradual Onset Continuous Performance Task^43^. As Participants were instructed to click a button when they saw pictures of a city (frequent stimulus) on the screen but withhold their response when a picture of a mountain appeared (infrequent target). Similar results were obtained in 6 participants who completed the task with reversed instructions (i.e., withheld responses to frequent stimuli and clicked a button when the infrequent target stimulus appeared). When participants made erroneous responses during infrequent target trials or a correct click response during the frequent target trials, power of higher frequency activity (HFA, aka., high gamma) was increased in the dorsal posterior Region I in sites whose y-coordinate corresponded to the location of sites whose stimulation caused symptoms in the hand region. Indicating that the activation of these sites during the experiment was due to button press. During the target stimulus, Region IV showed significantly stronger activation than all other regions. Red asterisks indicate statistical significance from ANOVA with post hoc permutation tests at both electrode and participant levels. Cohen’s d effect sizes for Region IV compared to all other Regions are displayed adjacent to the asterisks.

The majority of sites (87%; 81/93 electrodes) in the most anterior insular pole region (Region IV), encompassing cytoarchitectonic areas Id7, Id8, and Id10, were silent during electrical stimulation, compared with 59.8% in Region III and 41.8% and 29.8% in Regions II and I, respectively (Figure 4A). The few responsive sites in Region IV directly bordered Region III and were associated with sensations reported by the stimulation of Region III (visceral, 7.5% or anxiety-related sensations, 5.4%) - most likely reflecting inherent shift in electrode positions when location of electrodes were transferred from the native anatomical space to standard atlas space. Critically, we note that within each individual, the silent sites of the insula received equally strong electrical current compared to adjacent sites whose stimulation evoked reportable changes in the conscious state of the participant (responsive: median 3 mA, IQR 2–4 mA/ silent: median 4 mA, IQR 3–6) (Supplement Figure 1).

### Regionally and Functionally Specific Insular Activations During Experimental Condition of Salience Detection and Action Mode Decision

Guided by evidence from neuroimaging literature implicating the most anterior insula in large-scale salience^41^ and action-mode^42^ networks, we examined task-evoked electrophysiological dynamics of activity across insular recording sites during a Gradual Continuous Performance Task (GradCPT)^43^ that involves detection of salient (infrequent) stimuli during which a different mode of action (either go or no-go) was required. Experimental data were collected in 35 participants, 455 (240 left, 215 right, 210 posterior, and 245 anterior) insular electrodes.

We used the measure of high frequency activity (HFA, >70Hz) in each insular site to determine its engagement during different task conditions. During mouse clicks, selective increase of HFA was mostly localized to the posterior dorsal Region I (Figure 4B). The y-coordinates of these task-active sites overlapped with electrodes that elicited hand sensations during stimulation. During infrequent target trials, when the participants were asked to withhold from clicking, power of HFA increased in sites within Region IV (mean t-value = 5.3), significantly surpassing regions III (mean t-value = 2.0), II (mean t-value = −0.4), and I (mean t-value =−0.05) (one-way ANOVA: F= 79.55, p < 0.0001; all post-hoc electrode and participant level permutation tests p < 0.001 for Region IV vs. all other regions; Cohen’s d = 0.9–1.69, FDR corrected for multiple comparisons). Activated sites in Region III were concentrated near the boundary zone with region IV, again reflecting a possible shift in the actual position of the electrodes site due to transition from native anatomical to standard atlas space. We found similar results when the task instructions were reversed and participants were instructed to only respond with mouse clicks to infrequent stimuli and withhold responses to frequent stimuli (reverse GradCPT condition). This reverse condition, tested in a subset of 6 subjects with 100 electrodes, indicated that the increased activity in Region IV was not primarily due to response inhibition per se (Supplementary Figure 4).

We note that our claim of anatomical specificity of response profiles was quantitatively validated using two complementary approaches. First, we assessed deviations from spatial randomness by comparing region-wise response frequencies against null distributions generated by random sampling from the full insular electrode population. Second, we performed pairwise regional comparisons using binomial tests (Supplementary Figure 5 with statistics).

### Causal Electrophysiological Relationship Across Insular Regions

We are mindful that the effects of electrical stimulation cannot be assumed to be purely local, as injected currents always propagate from the site of stimulation to remote targets through anatomically determined networks. Consequently, behavioral and experiential effects elicited by stimulation may arise from downstream or distributed network activation rather than the engagement of the stimulated site itself. For this reason, the causal interpretation of prior stimulation findings from a region of interest must be evaluated within the context of the electrophysiological connectivity profile of that region.

To understand the intricate electrophysiological connections among insular regions, we relied on a proxy measure of causal interactions using the well-known method of cerebrocerebral evoked potentials (CCEPs). With this approach, about 40 single pulses of electrical pulses were delivered repeatedly (with each single pulse separated by 2 seconds from the next) to a pair of adjacent insular electrodes (~2mm edge to edge distance) in 47 participants (413 stimulated pairs, 209 left, 204 right, 236 posterior, 177 anterior) while the spectral changes in the electrophysiological local field potentials were captured in all other recording sites. In this analysis we relied on the analysis of ipsilateral sites (i.e., recordings were obtained in the same hemisphere where the repeated stimulations were applied (751 recording pairs, 385 left, 366 right, 449 posterior, 302 anterior). The recorded evoked signals were quantified using our recently developed approach where we did not solely rely on the power of evoked responses but also the consistency of evoked changes (i.e., intertrial phase coherence, ITPC) in the spectral field across all trials of stimulation (details in Method and in our recent publication^44^). With this approach, strong and consistent early responses evoked in the recording site suggested direct input from the stimulated site^45,46^, while jittered and delayed input often indicates indirect input (most likely via the thalamus^47^).

**Figure 5:**
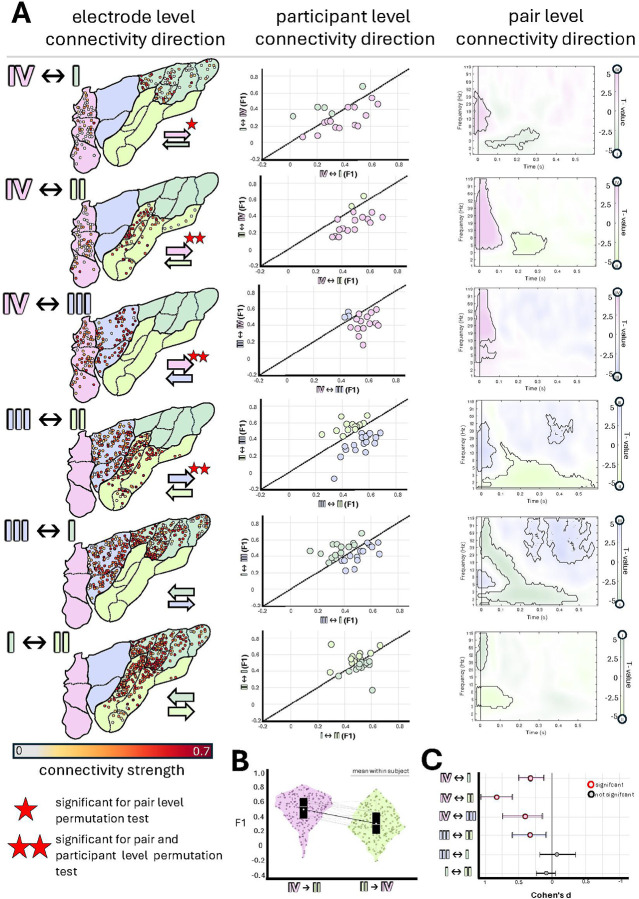
Cross Regional Asymmetric Connectivity. **A)** To investigate asymmetries in inter-regional connectivity, we examined bidirectional connectivity strength between insula regions. First, we visualized the spatial distribution of electrodes and connectivity strengths with each region serving alternately as source or target (left). Second, we plotted participant-level mean connectivity for both directions to identify directional preferences (middle). In these plots, each point represents one subject, and the identity line (diagonal) represents equal bidirectional connectivity. Points above or below this line indicate asymmetric connectivity strength favoring one direction over the other. For instance, most points fall below the diagonal line for Region IV to Regions II or IV to III while this asymmetry is not present for connectons between Regions I and II. Third, we performed spectrotemporal analysis to identify frequency-specific directional asymmetries across the broader spectrum of local field potentials (right). Color coding is according to the region showing greater connectivity in a given spectral field. For instance, Region IV demonstrated much stronger shift in the local field potentials across all other regions within 100ms – and in a broader range of frequencies (i.e., broadband) - particularly with respect to Regions II and III, shown in both the participant plot and the early high-frequency band in the spectrogram. By comparison, delayed and slow frequency connectivity from posterior region II to region IV indicated a possible indirect feedback mechanism. **B)** Violin plots for connectivity strength between Regions IV to II and II to IV, showing stronger connectivity from IV to II than the reverse not only at the group level, but also at the participant level (gray lines), with most participants showing a clear decrease in connectivity from Region II to IV compared to IV to II. **C)** Effect size with confidence interval for all regional comparisons. The asymmetry between regions IV and II showed the strongest overall effect size (Cohen’s *d*= 0.82, 95% CI [0.59, 1.05]).

To investigate the directionality and asymmetry of electrophysiological connections between regions, we relied on measures obtained in participants with electrodes in both regions of interest. Using three complementary analyses as detailed in Figure 5, our results revealed an asymmetric mode of causal electrophysiological connections across the four insular regions. We found bilateral connections across all insular regions documenting a clear inter-regional interaction. However, the connectivity profile was clearly asymmetrical (i.e., directional) across some of the pairs of regions, especially with Region IV exerting stronger “influence” on the other three regions compared to the afferent signals it received from those regions. For instance, the outflow from Region I to Region IV was weak (mean F1 = 0.24), while the reverse direction showed significantly stronger connectivity (mean F1 = 0.32; pair-level p = 0.007; Cohen’s d = 0.30, 95% CI [0.12, 0.49]). Region IV also exhibited significant directional asymmetry with Region II (mean F1: IV→II = 0.45, II→IV = 0.27; pair-level p = 0.0004, subject-level p = 0.0002; d = 0.82, 95% CI [0.59, 1.05]) and Region III (mean F1: IV→III = 0.53, III→IV = 0.43; pair-level p = 0.0085, subject-level p = 0.0009; d = 0.41, 95% CI [0.15, 0.74]). Subject-wise connectivity plots confirmed that Region IV outflow was stronger in the majority of individuals, and pair-level spectrograms revealed dominance of Region IV in early high-frequency responses (corresponding to N1 peak in classic CCEP terminology). The mid-anterior insula (Region III) demonstrated a smaller but also significant asymmetry only to Region II, with stronger connectivity in the IIIàII direction (mean F1: III→II = 0.51, II→III = 0.43; pair-level p = 0.01, subject-level p = 0.028; d = 0.32, 95% CI [0.09, 0.58]). While Region I received substantial direct input from Region IV, it maintained balanced bidirectional relationships with Regions II and III, with no significant asymmetry in overall connectivity strength (mean F1: I→II = 0.49, II→I = 0.47; I→III = 0.41, III→I = 0.39). However, pair-wise spectrogram analysis revealed frequency-dependent asymmetries: Regions I and II showed stronger influence over Region III in late low-frequency range, suggesting a possible feedback mechanism^47,48^. Notably, this low-frequency asymmetry was observed only between the posterior regions (I and II) and the mid-anterior Region III.

### Cortical Connections of Insular Regions

**Figure 6:**
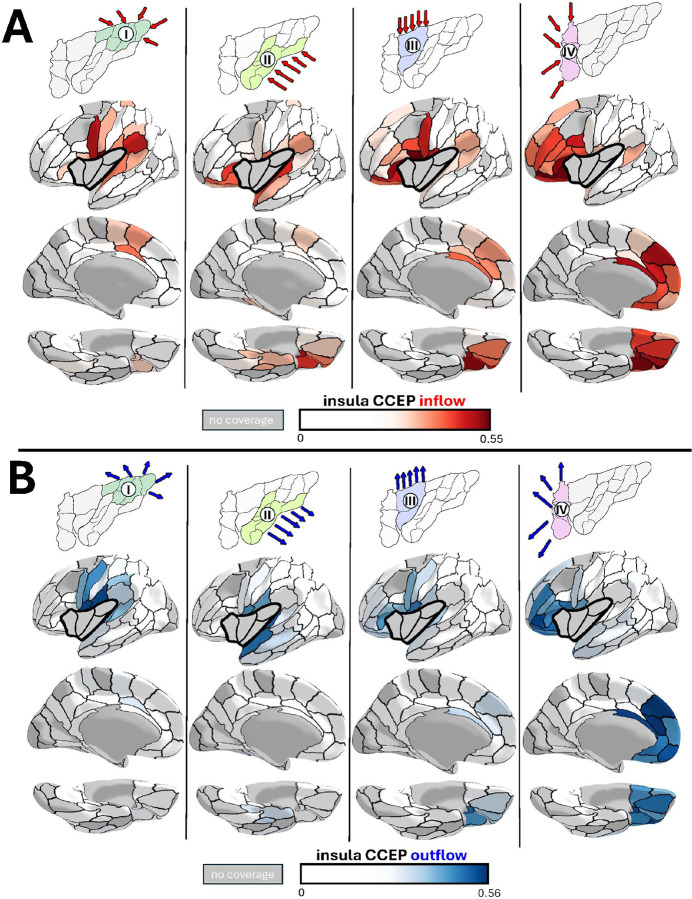
Whole-Brain Connectivity of Insular Regions. Connectivity strength (F1) between each insula region and available cortical regions of the Brainnetome Atlas with sufficient coverage (at least 3 participants) is color-coded from white to dark red (inflow) /dark blue (outflow). Cortical regions without sufficient coverage are depicted in gray. A) Inflow connectivity from cortical areas especially to insular regions II and III showed strong connectivity between region II/III and the directly bordering opercular and planum temporale sites. Regions III and IV received strong inflow from the orbitofrontal, prefrontal, and anterior cingulate cortex, with region IV showing substantially stronger connections than region III. B) Outflow connectivity from insula regions to cortical areas revealed strong coupling between region I and somatosensory cortices that was not observed in region II. Regions III and IV showed strong outflow connectivity to the orbitofrontal, prefrontal, and anterior cingulate cortex, with region IV again showing stronger connections than region III.

We next tested whether the structural and functional segregation within the insula reflects distinct connectivity profiles with cortical networks. Using bidirectional CCEPs approach in 56 participants, we systematically mapped causal connectivity by stimulating and recording from all available insular and cortical electrode pairs (Figure 6).

This bidirectional approach enabled us to distinguish insula outflow (insula à cortex) from inflow (cortex à insula), revealing whether insular regions preferentially receive information from, send signals to, or bidirectionally communicate with specific brain networks. The dataset comprised 460 insular stimulation pairs, 930 insular recording sites, 2,643 cortical stimulation pairs, and 5112 cortical recording sites, with balanced hemispheric (left/right) and anterior-posterior coverage. For each insular region, we computed mean connectivity strength to ipsilateral brain parcels of the multimodal Brainnetome Atlas^49^, a comprehensive whole-brain parcellation scheme based on anatomical landmarks and structural/functional connectivity. We included parcels represented in at least 3 participants for each insular region to ensure robust connectivity estimates, visualizing results on the surface-based fsaverage template.

The connectivity profiles revealed strong regional differentiation, especially between Regions IV and I. Region IV demonstrated robust bidirectional connectivity with orbitofrontal cortex (e.g. area OrG_6_5: inflow 0.44 ± 0.16, outflow 0.45 ± 0.17), ventrolateral prefrontal cortex (e.g. area MFG_7_4: inflow 0.44 ± 0.22, outflow 0.47 ± 0.22), and anterior cingulate cortex (e.g. area CG_7_3: inflow 0.47 ± 0.19, outflow 0.56 ± 0.14), while showing minimal connectivity with secondary somatosensory cortex (area IPL_6_3: inflow 0.14 ± 0.31, outflow 0.06 ± 0.25). Region I exhibited the opposite pattern: weak connectivity with orbitofrontal cortex (area OrG_6_5: inflow 0.09 ± 0.24, outflow 0.09 ± 0.24) limbic regions (e.g. area CG_7_3: inflow 0.07 ± 0.24, outflow 0.09 ± 0.24), but robust connections with secondary somatosensory cortex (area IPL_6_3: inflow 0.32 ± 0.26, outflow 0.36 ± 0.23).

Regions II (inflow) and III (inflow/outflow) exhibited connectivity patterns predominantly involving perisylvian structures—including the frontal, central, and parietal opercula, and planum temporale—as well as the orbitofrontal cortex. Region III additionally showed moderate prefrontal and limbic connectivity, though this was substantially weaker than Region IV across most parcels within these networks. Collectively, these data position Region IV within the prefrontal network including the anterior cingulate cortex while Region I is selectively related to somatosensory cortices.

## DISCUSSION

Using a multiprong intracranial mapping approach across a high number of neurosurgical participants, our findings reveal that the insular cortex is organized into a hierarchically structured, multipart system that is largely aligned with microstructural gradients. Posterior insular regions support somatosensory, in particular nociceptive, processing, with a finely organized somatotopic representation dorsally and a coarser, non-somatotopic body representation ventrally. Mid-anterior insula is causally linked to visceral sensations arising from the body core, bridging sensory and affective domains. The most anterior insula emerges as a hub, lacking direct access to subjective percepts, but exerting strong outbound influence across other insular networks and bilateral electrophysiological relationship with prefrontal, including the anterior cingulate, networks. Together, our findings establish the insula as a distributed, and yet tightly interconnected, multipart structure. Importantly, statistical validation of our data with rigorous null modeling and regional comparisons confirmed that each of the four insular regions is selectively engaged for distinct response profiles with distinct mode of electrophysiological connectivity with each other confirming the insula’s quadripartite functional architecture beyond a purely descriptive map.

We acknowledge that the quadripartite functional architecture of the insula described here is derived from recordings at hundreds of insular sites across several dozen participants. Although this sampling provides substantial coverage, it remains likely, and indeed expected, that future investigations employing higher spatial resolution and larger cohorts will reveal additional complexity beyond the four-part scheme proposed here. Our framework should therefore be viewed as constrained by the temporal and methodological parameters of the present study.

Nonetheless, within these constraints, our findings demonstrate a robust and reproducible functional partitioning of the insula, characterized by fine grained functional divisions (even including somatotopic sensory maps) with clearly asymmetric relationships (i.e., polar anterior “dominance”) among them. This functional organization extends beyond the classical dichotomous (posterior versus anterior) or cytoarchitectonic (granular, dysgranular, agranular) subdivisions, suggesting a more differentiated and functionally specific architecture than previously appreciated. In the following text, we highlight some of our findings that may provide valuable translational insights or relevant mechanistic information related to extant theoretical models of interoception and the role of insula in human awareness.

Our observation of somatotopic organization within dorsal posterior Region I is consistent with prior rodent studies^50^, as well as human neuroimaging^51,52^ and electrical stimulation^22^ reports. Moreover, the anatomical location of this somatotopic field closely corresponds to the region described in primate literature as the primary interoceptive cortex^5,6^. It further aligns with contemporary anatomical observations demonstrating a granular microstructure with a prominent layer IV, characteristic of primary sensory cortices. Together, these converging lines of evidence support the interpretation of dorsal posterior Region I as a primary interoceptive hub. These findings also suggest that future neuromodulation strategies targeting insular cortex for chronic pain- for example, focal facial pain - may benefit from anatomically informed targeting of this somatotopically organized subregion.

In contrast to Region I, the anterior Region IV showed no consistent evoked subjective effects and yet was characterized by distributed coupling with higher-order prefrontal association cortices. As such Region IV has the features commonly associated with transmodal integrative hubs^53^. In our prior work, we have demonstrated that the probability of eliciting subjective experiences with electrical stimulation decreases along a cortical gradient (highest in focal sensorimotor regions to lowest rates of eliciting response when highly distributed, integrative association networks were stimulated)^54^. The functional profile of Region IV aligns well with this principle, suggesting that its contribution to cognition may emerge from large-scale network interactions with prefrontal cortices and asymmetrically strong outbound connections to the more posterior insular regions - supporting allostatic regulation^55^ and active inference^56^ of bodily states.

These observations integrate disparate findings in the insula literature into a functionally and cytoarchitecturally grounded framework validated by experimental data from causal perturbation experiments. In particular, our results reconcile two seemingly distinct models of insular information flow: one proposing posterior-to-anterior dominant transmission^6^ and the other emphasizing anterior-toposterior directionality^8,9^. We identify direct posterior-to-anterior connections that may support the relay of interoceptive signals to anterior insular Region IV and thereby to higher-order cortical networks. At the same time, we observe a stronger anterior-to-posterior gradient of electrophysiological connectivity, consistent with the notion of “top-down” modulation and predictive regulation of bodily signals. Together, our findings also bridge these two prior models with the concept of “as-if body loops,”^57^ whereby higher-order cortical regions access representations of current and anticipated bodily states without relying exclusively on peripheral autonomic feedback^58^. Bidirectional interactions between prefrontal cortices and Region IV, which in turn has its asymmetric connectivity with other insular subregions, may provide a plausible anatomical and physiological substrate for this proposed mechanism.

Additionally, our findings are relevant for discussions about the insula serving as a core component of the action mode network (AMN), a large-scale brain network that supports the selection, initiation, and regulation of goal-directed behavior by translating salient internal and external information into action^42,59^. Based on our findings, Region IV could be seen as a hub for integrating motivational, cognitive, and motor-related signals to support adaptive behavior, particularly in contexts requiring rapid decision-making, effortful control, or behavioral flexibility while other insular regions may be associated with AMN, based on their role in providing a readout of bodily states as actions are being prepared or as a feedback of bodily state after they have been executed.

The asymmetric electrophysiological connectivity pattern we describe between Region IV and other insular regions may be clinically relevant for understanding the pathophysiology of chronic pain and anxiety disorders, as hypothesized in the extant literature^12,60^. Dysregulation between Region IV and Regions I/II or III could result in abnormally elevated baseline activity within these regions or excessive broadcasting of nociceptive or visceral signals to broader brain networks. Such signals, potentially shaped by prior experiences of pain or anxiety, may occur in the absence of ongoing harmful bodily states. Alternatively, a dysregulation in the relationship between Region IV and I/III may lead to heightened predictions of pain- or anxiety-provoking states, despite a lack of corresponding stimuli. In this framework, chronic anxiety may arise when Region IV over-weighs anticipated aversive bodily states, biasing downstream activity in Region III toward heightened visceral output, even in the absence of corresponding sensory input. Such a mismatch between predicted and actual interoceptive signals would generate sustained error signals, reinforcing hypervigilance and maladaptive affective responses.

We acknowledge that our observations were obtained from neurosurgical participants with focal epilepsy rather than healthy controls. Nevertheless, we believe these findings are generalizable to the broader population. Several lines of data support this claim. First, the insular cortex in the majority of our participants was radiologically normal and outside the seizure onset zone. Even in those with radiographically abnormal insular tissue, we have documented in a separate study that stimulation of pathological tissue elicits symptoms similar to those produced by stimulation of the same insular regions in individuals without structural abnormalities^29^. Second, conscious “auras” arising from seizures in a given brain region closely resemble the subjective experiences evoked by electrical stimulation of that region, suggesting that stimulation-induced states directly reflect conscious human experience^61^. Third, epileptic tissue behaves as physiologically normal tissue when not engaged in seizure activity or when the transient pathological epileptiform discharges are not present^62^. That said, we recognize that our observations are constrained by the clinical context in which they were obtained. Recordings were made using standard clinical electrodes, and both the number and placement of insular contacts in each participant were dictated by clinical considerations rather than experimental design. These limitations aside, we hope that our study will provide a novel framework of insular functional and structural architectural organization that integrates top-down and bottom-up processing. We have made our insular map available in the MNI space, which may provide a new foundation for future studies employing higher-resolution recordings and neuromodulation approaches targeting the human insula.

## ONLINE METHODS

### Participants and Electrode Implantation

The present study includes 87 participants (53 male, 34 female; age range 19–67 years) who underwent stereoelectroencephalography (SEEG) for seizure localization at Stanford University Medical Center. All participants provided informed consent under protocols approved by the Stanford Institutional Review Board. Electrode implantation sites were determined solely based on clinical criteria by a multidisciplinary team including specialists in epilepsy, neurosurgery, psychiatry, neuropsychology, and radiology. No electrodes were implanted solely for research purposes.

The extended data were acquired in a period spanning more than a decade. As noted, all experiments demonstrated balanced hemispheric laterality and anterior-posterior coverage sufficient for whole-insula mapping. Our stimulation cohort included a total of 475 depth electrodes (Ad-Tech Medical Instruments; 0.86 mm diameter contacts, 2.29 mm height, 3–5 mm center-to-center spacing) implanted across all participants. Insular implantation was preferentially performed using oblique trajectories through the insular cortex the safety of which has been previously documented^39^. This provided extensive insular coverage within each participant. Details on surgical procedures and research protocols are provided in our recent publications^63^.

### Electrode Localization and visualization

Electrode contacts were localized using the established iELVis workflow^64^. Each participant’s preoperative T1-weighted MRI was first preprocessed with FreeSurfer l^65^ to enhance image contrast, reconstruct cortical surfaces in native space, and align them to the fsaverage template. The preprocessed MRI was then co-registered to the postoperative CT scan using tools from the FMRIB Software Library (FSL)^66^, enabling precise electrode mapping within each participant’s brain space. Electrode contacts were manually identified in BioImageSuite^67^ by detecting the characteristic bright signals at electrode locations on the fused MRI/CT images and verifying their alignment with known surgical trajectories. The coordinates of each electrode contact were automatically extracted in native voxel space, FreeSurfer surface space, and MNI-152 space using the iELVis toolbox.

The anatomical localization of electrodes was determined using a two-step approach. First, a trained anatomist (J.P.) identified the location of each electrodes based on macroscopic anatomical landmarks in the native anatomical space, and labeled those within the insular boundaries, as “insula”. Second, the microstructural subdivision of the human insula from the Julich Brain Atlas^4,35–38,68,69^ was co-registered from MNI-152 space to each participant’s T1-weighted native space using ANTs providing higher-resolution localization beyond macroscopic boundaries. The coordinates of each Insula electrode contact were then used to automatically extract the corresponding atlas label. For insula contacts, microanatomical labeling was additionally reviewed and verified by another anatomist and specialist in the cytoarchitectonic map of the insula (J.Q.) through direct inspection of the participant’s T1-weighted images in combination with the co-registered atlas. Insula electrodes were visualized on a flat map of the microstructural human insula parcellation.

### Experiments and Analysis

#### Functional Mapping of Insula with Intracranial Electrical stimulation

Intracranial electrical stimulation of the insula was performed as part of routine clinical evaluation during presurgical epilepsy monitoring. Bipolar stimulation was delivered between pairs of adjacent electrode contacts using a Nihon Kohden cortical stimulator (MS-120BK-EEG) with PE-210AK switchbox, interfaced with Nihon Kohden video-EEG monitoring equipment (WEE-1200, 1000-Hz sampling rate). Stimulation consisted of alternating square-wave pulses at 50 Hz frequency with 0.2 ms pulse width, delivered for 1–4 seconds per trial. Current amplitude was systematically increased but remained below the threshold for producing after-discharges, which was determined individually for each electrode pair. The complete distribution of current amplitudes used across all stimulation sites is presented in Supplementary Figure 1.

To ensure methodological rigor and minimize bias, we implemented a comprehensive protocol with multiple safeguards. The participant, seated upright in bed, faced a camera with no investigators in their direct line of sight, preventing inadvertent cueing through facial expressions or body language. All stimulation parameters, including electrode location, current amplitude, frequency, and sham versus active trials, were configured silently keeping the participant blind to conditions. The stimulator’s built-in sham function delivered indistinguishable 0-mA trials randomly interspersed throughout each session. Stimulation-evoked responses were considered valid only if they met the following stringent criteria: (1) were reproducible only during real, but not sham, stimulations, (2) were reproducible when the same site was stimulated multiple times, and (3) exhibited a clear dose–response relationship (i.e., effect intensity increasing as stimulation intensity increased).

During each trial, the neurologist monitored the computer while a second observer documented stimulation parameters and preliminary keywords describing participant responses. Participants were instructed to report any subjective experiences or sensations, with all interactions captured on synchronized audio-video recordings. An external rater naive to study aims and stimulation parameters transcribed the recordings verbatim. Final transcripts were cross-referenced against preliminary bedside notes, with discrepancies resolved through detailed review of archived recordings. On this basis, each contact was assigned to a distinct functional category: somatosensory (tingling, numbness, or pressure sensations), pain and/or temperature (noxious or heat sensations occurring either separately or together), chemical perception (taste and olfaction), visceral (internal bodily sensations of nausea, dizziness, wave-like energy in the body core, throat or head), emotional (primarily anxiety and nervousness), and non-responsive (no subjective change experienced upon stimulation). Additionally, somatosensory and pain/temperature responses were categorized according to their anatomical localization (head, face, mouth, trunk, back, arms, legs), possible combinations between body parts, and laterality (left, right, bilateral).

#### Functional Clustering of Insular Microstructural Areas

Next, we performed unsupervised clustering of insular microstructural areas based on their functional response profiles during electrical stimulation. For each area, we calculated the frequency of four functional features across all electrode pairs: visceral responses, somatosensory responses, pain/temperature sensations, absence of responses. Features with <5% frequency (chemical perception: 2.3%, anxiety: 1.8%) were excluded to avoid overfitting and ensure clustering was driven by robust, replicable functional patterns rather than rare, potentially idiosyncratic observations. We standardized feature vectors and applied K-means clustering with cluster number optimization using the elbow method. For visualization, we performed multidimensional scaling (MDS) to project the high-dimensional feature space into 3D Euclidean space enabling spatial interpretation of functional similarity between areas. We calculated stress as a goodness-of-fit measure between the resulting MDS configuration and the underlying data; quantifying how well pairwise distances are preserved in the lower-dimensional representation.

#### Somatosensory Evoked Potentials (SEPs)

To validate the somatotopic organization of somatosensory and pain responses identified through direct cortical stimulation mapping, we analyzed somatosensory evoked potentials (SEPs) from an independent Italian cohort of 37 participants recorded from 218 electrodes in the insula implanted at Ospedale Niguarda, Milan, Italy, following institutional ethics approval (ID 939–2.12.2013). Participants underwent peripheral nerve stimulation (100 pulses, 0.2 ms duration, 1 Hz) targeting the median nerve (hand), tibial nerve (foot), and mandibular branch of the trigeminal nerve (face) using a Nihon Kohden Neuropack M1 MEB-9200 system. Recordings were obtained from the contralateral hemisphere.

To ensure adequate stimulation of the target nerve, the motor threshold was first determined by observing characteristic motor responses, confirming correct nerve localization. Stimulation intensity was then set at 110% motor threshold (range: 6.9–15.2 mA for tibial, 3.5–6.4 mA for median, 2.6–5.2 mA for trigeminal) to ensure that the participant remained above motor threshold during the stimulation.

Electrode localization followed the same MRI/CT fusion and FreeSurfer-based workflow as described above. Insular electrodes were mapped to the microstructural insula flat-map template. Statistical significance for each insular electrode and stimulated nerve was determined by analyzing the phasic component (significant activity within 70 ms after stimulation).

Statistical significance was assessed by quantifying stimulus-induced changes in gamma-band activity. Signals from all contacts located in the insular grey matter were segmented from −250 to 750 ms relative to stimulus onset and decomposed using time–frequency analysis. Gamma-band power (50–150 Hz) was extracted in non-overlapping 5-ms time bins across equispaced 5-Hz frequency bands. For each time–frequency bin, post-stimulus gamma power was statistically compared against the zero-mean using a one-tailed test (Bonferroni corrected) to specifically capture stimulus-related power increases. Responsiveness was determined by quantifying the number of significant gamma-band time bins within the phasic intervals: 10–50 ms for median nerve stimulation, 5–55 ms for trigeminal nerve stimulation, and 30–70 ms for tibial nerve stimulation (see Avanzini et al, 2018). Electrodes were labeled as significant when the significant bin count exceeded 2. To compare somatotopic organization between peripheral nerve stimulation and direct cortical stimulation, we analyzed the y-coordinates of significant electrodes for each specific body region relative to the y-coordinates of electrodes evoking corresponding sensations during direct cortical stimulation.

#### Gradual-Onset Continuous Performance Task (gradCPT)

We tested whether insular sites were engaged during a task that is known to require salient infrequent stimulus detection and change of action mode. We recorded neural activity from 35 participants across 455 insular electrodes during a gradual-onset continuous performance task^70^. The task was administered at bedside via laptop using Psychophysics Toolbox in MATLAB. Grayscale images of city or mountain scenes gradually transitioned over 800 ms at a 90% city (frequent non-target) to 10% mountain (infrequent target) ratio, randomized for each run. Participants were instructed to press the spacebar for city scenes and withhold responses for mountain scenes. Each participant completed multiple runs (range: 2–12), with each run lasting 2–8 min. TTL trigger pulses marked stimulus onset times on a dedicated EEG channel.

Continuous intracranial EEG was recorded during task performance (Tucker-Davis system, sampling rate: 1000 Hz). The data were preprocessed following a previously established pipeline (Stieger et al., 2024): First, notch filters with center frequency at 60, 120, and 180 Hz were applied to remove power line noise. Then, common average re-referencing was applied, with channels excluded from the reference if they: (a) showed variance exceeding five times or falling below one-fifth of the cross-channel median variance; (b) contained spikes with a rate exceeding 1 spike per second, where spikes were defined as inter-sample voltage changes ≥ 80 μV; (c) exhibited pathological activity with pathological high-frequency oscillations (Liu & Parvizi, 2019); or (d) displayed abnormal power spectral characteristics upon visual inspection. Finally, we performed time-frequency decomposition on each re-referenced signal, by applying a continuous Morlet wavelet transform logarithmically spaced between 1 and 256 Hz.

To identify target-responsive electrodes within the insular cortex, we compared high-frequency activity (HFA; 70–170 Hz) power between two specific conditions: target trials (correct omissions to rare mountain scenes) and baseline trials (correct commissions to frequent city scenes). Following established GradCPT analytic protocols^71^, HFA power time courses were smoothed, epoched, and subsampled to avoid potential contamination with responses evoked by rare mountain scenes and to ensure an equal number of target and baseline trials per subject. To determine statistical significance, trials were pooled across all runs for each participant. We conducted independent-samples t-tests to compare the mean HFA power amplitude between target and baseline trials within a specific temporal window of 400–1200 ms post-stimulus (relative to the onset of the image fade-in). Electrodes were classified as target-responsive if the difference in mean HFA power between these two conditions reached a two-tailed significance threshold of p < 0.05, after False Discovery Rate (FDR) correction was applied to account for the total number of electrodes within each subject. A similar contrast was employed to identify click-responsive electrodes. For this analysis, we compared HFA power during all commission trials (click responses to either city or mountain scenes) against all omission trials (withhold responses), regardless of stimulus category.

To assess regional differences in salience task-related activity, we performed a one-way ANOVA on electrode-level t-values across the four clusters, followed by post-hoc permutation tests for all between-cluster comparisons (10,000 iterations with randomly shuffled cluster labels). Outliers were detected using combined IQR and z-score methods (identifying values falling below Q1 – 1.5 × IQR or above Q3 + 1.5 × IQR, and with |z| > 3). Multiple comparisons were corrected using False Discovery Rate (FDR, Benjamini-Hochberg, α = 0.05). Effect sizes were quantified using Cohen’s d with 95% confidence intervals estimated via bootstrap resampling (10,000 iterations) of electrodes within each cluster. To control for inter-subject variability, we additionally conducted subject-level permutation tests by aggregating electrode t-values per participant within each cluster, then randomly flipping the sign of within-participant cluster differences to create null distributions under the hypothesis of no systematic effect. Effects were considered robust if they achieved FDR-corrected significance (p < 0.05) at both electrode and participant levels, with 95% confidence intervals for effect sizes excluding zero in all regional comparisons.

#### Statistical validation of regional response profiles

Building on the previous experimental approach in combination with a high-resolution microstructural atlas of the insula, a pattern of four distinct insular regions emerged with unique functional and structural properties. To validate the anatomical specificity of response profiles in each region (pain/temperature, somatosensory, visceral, anxiety, salience, body-part representation, and no response to stimulation), we employed two complementary statistical approaches. Given the nominal quality of our electrical stimulation data and binary salience task activation data (activated/not activated), we used binomial testing and frequency comparisons of regional activation against population baselines, with global correction for multiple comparisons.

First, for each region, we tested whether observed response frequencies deviated from spatial randomness using two-sided binomial tests. The null hypothesis assumed that responses within each region represented random samples from the overall population distribution, independent of anatomical localization. To simulate this null hypothesis, we generated a bootstrap distribution for each region-response combination through 10,000 iterations of random resampling with replacement. Each iteration randomly sampled electrodes and their respective responses equal to the regional sample size from the entire population. The resulting bootstrap distribution for each response type was centered on the population frequency, providing the expected value under spatial randomness against which we compared observed regional proportions. We visualized each region-category combination with the bootstrap-derived null distribution (kernel density estimation), population frequency and observed regional frequency (Figure 5).

Second, to directly compare response profiles between regions, we performed targeted one-sided binomial tests for specific hypothesized regional specializations: Region I enrichment for pain/temperature and single body-part responses versus Regions II–IV; Region II enrichment for somatosensory and multiple body-part responses versus other regions; Region III enrichment for visceral and anxiety responses versus other regions; Region IV enrichment for salience activation and no response to electrical stimulation versus other regions. For each comparison, the frequency in the comparison region served as the null probability.

All p-values across both analyses (56 total tests) were pooled and corrected using the Benjamini-Hochberg false discovery rate (FDR) procedure at α = 0.05, controlling family-wise error across the entire statistical framework. Results were considered significant only after FDR correction. Statistical analyses were performed in Python using scipy.stats.

#### Cerebro- cerebral evoked potentials – within insula connectivity

We analyzed causal connectivity both within the insula and between insular regions and other cortical areas using cerebro-cerebral evoked potentials (CCEPs), a method we have extended from cortico-cortical evoked potentials to include subcortical structures as described in our previous work^72^. Unlike conventional CCEP approaches that rely on arbitrary amplitude thresholds or canonical N1/N2 peak identification, our method quantifies the full spectrotemporal richness of evoked local field potentials, recognizing that evoked signals often deviate from canonical waveforms.

Single electrical pulses were administered at 0.5 Hz in a bipolar configuration, stimulating electrode pairs while recording from all other electrodes. iEEG data were acquired at 1000 Hz sampling rate. Preprocessing included notch filtering at 60, 120, and 180 Hz, followed by exclusion of noisy channels and trials. Noisy channels were identified based on extreme raw amplitude (>5 SD), frequent spikes (>3× median spike rate), high mean absolute voltage (>4 SD), or elevated trial variance (>3.5 SD). Trials with mean amplitudes exceeding 4 standard deviations were also discarded. Time-frequency analysis was performed using Morlet wavelet decomposition with frequencies log-spaced from 1–256 Hz (5 cycles per wavelet), generating instantaneous power and phase data downsampled to 200 Hz. Power spectrograms were baseline-corrected, log-transformed, and z-scored across time and frequency. Phase consistency across trials was quantified using inter-trial phase coherence (ITPC), which was square-root transformed and z-scored. Connectivity strength was measured using an in-house pipeline based on power spectrum and ITPC features. Specifically, we quantified the F1 feature, which appears as an early sharp wave with increased high-gamma power and tight phase-locking to stimulation onset, comparable to the traditional N1 component and indicative of feedforward connectivity. F1 was detected using sliding-window cross-correlation to compare normalized power and ITPC with predefined group-derived templates. Connectivity strength was defined as the peak correlation coefficient (peak_maxCor) across time, identified using MATLAB’s findpeaks function.

For the within-insula analysis, we examined CCEP-derived connectivity (F1 scores) from 47 participants with 413 stimulation and 751 recoding pairs, restricting our analysis to ipsilateral stimulation-recording sites (i.e., stimulation and recording sites within the same hemisphere). This analysis addressed two complementary questions: first, the overall strength of connectivity between insular subregions, and second, whether these connections exhibited directional asymmetry, with stronger connectivity in one direction compared to the reverse.

To ensure data quality while preserving biological variability, we applied a conservative dualcriterion outlier detection approach combining interquartile range (IQR) and z-score methods. For each connectivity distribution, we first identified potential outliers using the IQR method: values falling below Q1 − 1.5 × IQR or above Q3 + 1.5 × IQR, where Q1 and Q3 represent the 25th and 75th percentiles, respectively, and IQR = Q3 − Q1. We then applied a complementary z-score criterion, flagging values with |z| > 3 (i.e., more than 3 standard deviations from the mean). Only data points identified as outliers by both methods were excluded, minimizing the risk of removing true neurophysiological variability while eliminating technical artifacts or recording errors. Following outlier removal, we excluded contralateral stimulation-recording pairs and restricted all subsequent analyses to ipsilateral hemisphere connections to ensure anatomically interpretable directionality.

Next, we employed three complementary analytical approaches at different levels of data aggregation. First, at the individual electrode level, we visualized the spatial distribution of directional connectivity by projecting all individual electrodes for each regional comparison onto the insula template, with each electrode color-coded by its mean connectivity strength (F1 score). For each recording electrode, connectivity strength was averaged across all stimulation sites in the source region, and then this process was reversed to visualize connectivity in the opposite direction.

Second, at the pair-level, we identified matched electrode pairs (i.e., the same two electrodes stimulated in both directions) within each subject, yielding bidirectional connectivity measurements. To test for directional asymmetry while accounting for the non-independence of multiple electrode pairs within participants, we used permutation testing (10,000 iterations). On each iteration, we randomly flipped the direction labels (forward ↔ backward) for all pairs from each participant together as a unit, then calculated the mean difference across all pairs for that permuted dataset. This process generated a null distribution comprising 10,000 mean differences, representing the range of values expected by chance in the absence of true directional asymmetry. The observed mean difference was considered statistically significant (p < 0.05) if it was more extreme than 95% of the values in this null distribution. Effect sizes were quantified using Cohen’s d for paired samples at the electrode-pair level. To estimate how much our Cohen’s d values vary due to sampling variability while accounting for the non-independence of electrode pairs within participants, we used subject-clustered bootstrap resampling (10,000 iterations). On each iteration, we randomly resampled participants with replacement (selecting the same number of participants as in the original dataset), included all electrode pairs from each selected subject, and recalculated Cohen’s d from this resampled dataset. This process generated a distribution of 10,000 Cohen’s d values representing the sampling variability of our effect size estimate, from which we extracted the 2.5th and 97.5th percentiles as the 95% confidence interval bounds.

To examine directional asymmetries across the full time-frequency spectrum beyond the early high-frequency F1 response, we calculated contrast spectrograms between paired electrodes for each regional comparison. For matched electrode pairs, power spectrograms were computed in each direction, averaged across pairs and trials, and subtracted to yield directional contrast maps (forward minus backward). Cluster-based permutation testing identified statistically significant spectrotemporal clusters. Candidate clusters were defined as contiguous time-frequency regions where point-wise t-tests across electrode pairs showed significant directional differences (α = 0.05). The sum of t-values within each cluster was computed. Significance was assessed via 5,000 permutations where direction labels were randomly shuffled, contrast spectrograms recalculated, and maximum/minimum cluster t-sums recorded to form a null distribution. Observed clusters exceeding 95% of the null distribution were deemed significant.

Third, as a sensitivity analysis, we conducted subject-level analyses by averaging all pairs within each participant to yield one bidirectional comparison per participant (removing the clustering issue). For each subject, we calculated the mean forward value and mean backward value across all electrodes. Statistical significance was assessed using paired permutation tests (10,000 iterations). On each iteration, we randomly flipped the direction labels (forward ↔ backward) for each participant independently, then calculated the mean difference across participants for that permuted dataset. This generated a null distribution of 10,000 mean differences, and the observed mean difference was considered significant (p < 0.05) if it was more extreme than 95% of the permuted values. Results were visualized as scatter plots of mean forward versus backward connectivity for each participant against the line of equality (representing symmetric connectivity), where deviations from this line indicated asymmetric connectivity.

Results were corrected for Multiple comparisons using the Benjamini-Hochberg false discovery rate method across all directional comparisons within each analysis strep. All analyses were performed in Python 3.x using NumPy, SciPy, pandas, statsmodels, matplotlib, and seaborn.

#### Cerebro- Cerebral Evoked Potentials – Insula-Cortical Connectivity

Using the same methodology as described above, we next investigated connectivity patterns and potential differences between the four discovered insular regions and the entire cortex across 56 participants with 460 insula stimulation and 930 recording pairs and 2643 cortical stimulation and 5112 recording pairs. In addition to Julich-Brain atlas that provides complete coverage of the insula, we employed the multimodal Brainnetome^49^ atlas for comprehensive cortical parcellation.

After outlier detection using combined IQR and z-score methods (see above), we excluded contralateral stimulations and restricted our analysis to ipsilateral hemisphere connections. We included only Brainnetome parcels that contained electrodes from at least 3 participants for each of the four insular regions, ensuring comparable sampling across regions. The respective outflow (insula → cortical parcel) and inflow (cortical parcel → insula) color-coded mean connectivity values were visualized on the fsaverage template.

## Supplementary Material

Supplementary Files

This is a list of supplementary files associated with this preprint. Click to download.

- SupFig4.pdf
- SupFig3.pdf
- SupFig1.pdf
- SupFig2.pdf
- SupFig5.pdf

## Data Availability

We have mapped the four insular regions in MNI152 ICBM 2009c Nonlinear Asymmetric space and will make it publicly available for download upon publication of our work. Electrophysiological connectivity data from our study along with the exact coordinates of the stimulated or recorded sites in MNI space will also be shared upon publication.

## References

[1] Sherrington C. S. The Integrative Action of the Nervous System. 317 (YALE UNIVERSITY PRESS, 1906).

[2] Namkung H., Kim S. H. & Sawa A. The Insula: An Underestimated Brain Area in Clinical Neuroscience, Psychiatry, and Neurology. Trends Neurosci 40, 200–207 (2017). 10.1016/j.tins.2017.02.002 28314446 PMC5538352

[3] ddin L. Q., Nomi J. S., Hébert-Seropian B., Ghaziri J. & Boucher O. Structure and Function of the Human Insula. J Clin Neurophysiol 34, 300–306 (2017). 10.1097/WNP.0000000000000377 28644199 PMC6032992

[4] Quabs J., Bittner N. & Caspers S. Structural Connectivity Differences Reflect Microstructural Heterogeneity of the Human Insular Cortex. Hum Brain Mapp 46, e70231 (2025). 10.1002/hbm.70231 40396764 PMC12093499

[5] Craig A. D. An ascending general homeostatic afferent pathway originating in lamina I. [Review] [78 refs]. Progress in Brain Research 107, 225–242 (1996). 8782522 10.1016/s0079-6123(08)61867-1

[6] Craig A. D. How do you feel--now? The anterior insula and human awareness. Nat Rev Neurosci 10, 59–70 (2009). 10.1038/nrn2555 19096369

[7] Damasio A., Damasio H. & Tranel D. Persistence of feelings and sentience after bilateral damage of the insula. Cereb Cortex 23, 833–846 (2013). 10.1093/cercor/bhs077 22473895 PMC3657385

[8] Seth A. K. Interoceptive inference, emotion, and the embodied self. Trends Cogn Sci 17, 565–573 (2013). 10.1016/j.tics.2013.09.007 24126130

[9] Barrett L. F., Mesquita B., Ochsner K. N. & Gross J. J. The experience of emotion. Annu Rev Psychol 58, 373–403 (2007). 10.1146/annurev.psych.58.110405.085709 17002554 PMC1934613

[10] Barrett L. F. & Simmons W. K. Interoceptive predictions in the brain. Nat Rev Neurosci 16, 419–429 (2015). 10.1038/nrn3950 26016744 PMC4731102

[11] Singer T., Critchley H. D. & Preuschoff K. A common role of insula in feelings, empathy and uncertainty. Trends Cogn Sci 13, 334–340 (2009). 10.1016/j.tics.2009.05.001 19643659

[12] Paulus M. P. & Stein M. B. An insular view of anxiety. Biol Psychiatry 60, 383–387 (2006). 10.1016/j.biopsych.2006.03.042 16780813

[13] Ibanez A., Gleichgerrcht E. & Manes F. Clinical effects of insular damage in humans. Brain Struct Funct 214, 397–410 (2010). 10.1007/s00429-010-0256-y 20512375

[14] Jones C. L., Ward J. & Critchley H. D. The neuropsychological impact of insular cortex lesions. J Neurol Neurosurg Psychiatry 81, 611–618 (2010). 10.1136/jnnp.2009.193672 20522870

[15] Ostrowsky K. Representation of pain and somatic sensation in the human insula: a study of responses to direct electrical cortical stimulation. Cereb Cortex 12, 376–385 (2002). 10.1093/cercor/12.4.376 11884353

[16] Ostrowsky K. Functional mapping of the insular cortex: clinical implication in temporal lobe epilepsy. Epilepsia 41, 681–686 (2000). 10840399 10.1111/j.1528-1157.2000.tb00228.x

[17] Isnard J., Guenot M., Sindou M. & Mauguiere F. Clinical manifestations of insular lobe seizures: a stereo-electroencephalographic study. Epilepsia 45, 1079–1090 (2004). 10.1111/j.0013-9580.2004.68903.xEPI68903 [pii] 15329073

[18] Nguyen D. K. Revisiting the role of the insula in refractory partial epilepsy. Epilepsia 50, 510–520 (2009). 10.1111/j.1528-1167.2008.01758.x 18717706

[19] Mazzola L. Gustatory and olfactory responses to stimulation of the human insula. Ann Neurol 82, 360–370 (2017). 10.1002/ana.25010 28796326

[20] Mazzola L., Mauguiere F. & Isnard J. Functional mapping of the human insula: Data from electrical stimulations. Rev Neurol (Paris) 175, 150–156 (2019). 10.1016/j.neurol.2018.12.003 30827578

[21] Mazzola L. Vestibular responses to direct stimulation of the human insular cortex. Ann Neurol 76, 609–619 (2014). 10.1002/ana.24252 25142204

[22] Mazzola L., Isnard J., Peyron R., Guenot M. & Mauguiere F. Somatotopic organization of pain responses to direct electrical stimulation of the human insular cortex. Pain (2009). https://doi.org/S0304-3959(09)00397-2 [pii] 10.1016/j.pain.2009.07.014 19665303

[23] Mazzola L., Faillenot I., Barral F. G., Mauguière F. & Peyron R. Spatial segregation of somato-sensory and pain activations in the human operculo-insular cortex. NeuroImage 60, 409–418 (2012). 10.1016/j.neuroimage.2011.12.072 22245639

[24] Afif A., Minotti L., Kahane P. & Hoffmann D. Anatomofunctional organization of the insular cortex: a study using intracerebral electrical stimulation in epileptic patients. Epilepsia 51, 2305–2315 (2010). 10.1111/j.1528-1167.2010.02755.x 20946128

[25] Stephani C., Fernandez-Baca Vaca G., Maciunas R., Koubeissi M. & Luders H. O. Functional neuroanatomy of the insular lobe. Brain Struct Funct 216, 137–149 (2011). 10.1007/s00429-010-0296-3 21153903 PMC3097350

[26] Pugnaghi M. Features of somatosensory manifestations induced by intracranial electrical stimulations of the human insula. Clin Neurophysiol 122, 2049–2058 (2011). 10.1016/j.clinph.2011.03.013 21493128

[27] Yih J., Beam D. E., Fox K. C. R. & Parvizi J. Intensity of affective experience is modulated by magnitude of intracranial electrical stimulation in human orbitofrontal, cingulate and insular cortices. Soc Cogn Affect Neurosci 14, 339–351 (2019). 10.1093/scan/nsz015 30843590 PMC6537947

[28] Penfield W. & Faulk M. E. Vol. 78 445–470 (Oxford Academic, 1955). 10.1093/brain/78.4.445 13293263

[29] Duong A. Topographical map of subjective states evoked by focal seizures and electrical stimulation of the human insula. Epilepsia (2025). 10.1111/epi.18433 40357762

[30] Duong A. Subjective states induced by intracranial electrical stimulation matches the cytoarchitectonic organization of the human insula. Brain Stimul 16, 1653–1665 (2023). 10.1016/j.brs.2023.11.001 37949296 PMC10893903

[31] Bartolomei F. The role of the dorsal anterior insula in ecstatic sensation revealed by direct electrical brain stimulation. Brain Stimul 12, 1121–1126 (2019). 10.1016/j.brs.2019.06.005 31196836

[32] Mesulam M. M. & Mufson E. J. Insula of the old world monkey. I. Architectonics in the insulo-orbito-temporal component of the paralimbic brain. The Journal of comparative neurology 212, 1–22 (1982). 10.1002/cne.902120102 7174905

[33] Bonthius D. J., Solodkin A. & Van Hoesen G. W. Pathology of the insular cortex in Alzheimer disease depends on cortical architecture. J Neuropathol Exp Neurol 64, 910–922 (2005). 10.1097/01.jnen.0000182983.87106.d1 16215463

[34] Amunts K. & Zilles K. Architectonic Mapping of the Human Brain beyond Brodmann. Neuron 88, 1086–1107 (2015). 10.1016/j.neuron.2015.12.001 26687219

[35] Quabs J. Cytoarchitecture, probability maps and segregation of the human insula. NeuroImage 260, 119453 (2022). 10.1016/j.neuroimage.2022.119453 35809885

[36] Grodzinsky Y. Logical negation mapped onto the brain. Brain Struct Funct 225, 19–31 (2020). 10.1007/s00429-019-01975-w 31680213 PMC6957563

[37] Hein M., Mohlberg H., Bludau S., Quabs J. & Amunts K. Probabilistic cytoarchitectonic map of Areas Ia2, Ia3, Id8, Id9, Id10 (v4.0). EBRAINS (2021). Id8: 10.25493/7QE6-PBE

[38] Kurth F. Cytoarchitecture and probabilistic maps of the human posterior insular cortex. Cereb Cortex 20, 1448–1461 (2010). 10.1093/cercor/bhp208 19822572 PMC2871375

[39] Afif A., Chabardes S., Minotti L., Kahane P. & Hoffmann D. Safety and usefulness of insular depth electrodes implanted via an oblique approach in patients with epilepsy. Neurosurgery 62, ONS471–479; discussion 479–480 (2008). 10.1227/01.neu.0000326037.62337.80 18596531

[40] Avanzini P., Pelliccia V., Lo Russo G., Orban G. A. & Rizzolatti G. Multiple time courses of somatosensory responses in human cortex. NeuroImage 169, 212–226 (2018). 10.1016/j.neuroimage.2017.12.037 29248698 PMC5864517

[41] Seeley W. W. The Salience Network: A Neural System for Perceiving and Responding to Homeostatic Demands. J Neurosci 39, 9878–9882 (2019). 10.1523/JNEUROSCI.1138-17.2019 31676604 PMC6978945

[42] Badke D’Andrea C. Action-mode subnetworks for decision-making, action control, and feedback. Proc Natl Acad Sci U S A 122, e2502021122 (2025). 10.1073/pnas.2502021122 40587801 PMC12260544

[43] Rosenberg M., Noonan S., DeGutis J. & Esterman M. Sustaining visual attention in the face of distraction: a novel gradual-onset continuous performance task. Atten Percept Psychophys 75, 426–439 (2013). 10.3758/s13414-012-0413-x 23299180

[44] Lyu D. Mapping human thalamocortical connectivity with electrical stimulation and recording. Nature neuroscience 28 (2025). 10.1038/s41593-025-02009-x PMC12664536 40664975

[45] Matsumoto R. Functional connectivity in the human language system: a cortico-cortical evoked potential study. Brain 127, 2316–2330 (2004). 15269116 10.1093/brain/awh246

[46] Keller C. J. Mapping human brain networks with cortico-cortical evoked potentials. Philos Trans R Soc Lond B Biol Sci 369 (2014). 10.1098/rstb.2013.0528 PMC4150303 25180306

[47] Russo S. Thalamic feedback shapes brain responses evoked by cortical stimulation in mice and humans. Nat Commun 16, 3627 (2025). 10.1038/s41467-025-58717-2 40240330 PMC12003640

[48] Parvizi J. Thalamus, evoked responses and triphasic waves. Nat Commun 16, 5221 (2025). 10.1038/s41467-025-60452-7 40473622 PMC12141664

[49] Fan L. The Human Brainnetome Atlas: A New Brain Atlas Based on Connectional Architecture. Cerebral Cortex 26 (2016). 10.1093/cercor/bhw157 PMC4961028 27230218

[50] Vestergaard M., Carta M., Guney G. & Poulet J. F. A. The cellular coding of temperature in the mammalian cortex. Nature 614, 725–731 (2023). 10.1038/s41586-023-05705-5 36755097 PMC9946826

[51] Henderson L. A., Gandevia S. C. & Macefield V. G. Somatotopic organization of the processing of muscle and cutaneous pain in the left and right insula cortex: a single-trial fMRI study. Pain 128, 20–30 (2007). 10.1016/j.pain.2006.08.013 17011704

[52] Brooks J. C., Zambreanu L., Godinez A., Craig A. D. & Tracey I. Somatotopic organisation of the human insula to painful heat studied with high resolution functional imaging. NeuroImage 27, 201–209 (2005). 10.1016/j.neuroimage.2005.03.041 15921935

[53] Mesulam M. M. From sensation to cognition. Brain 121 (Pt 6), 1013–1052 (1998). 9648540 10.1093/brain/121.6.1013

[54] Fox K. C. R. Intrinsic network architecture predicts the effects elicited by intracranial electrical stimulation of the human brain. Nature Human Behaviour 4, 1039–1052 (2020). 10.1038/s41562-020-0910-1 PMC7572705 32632334

[55] Sterling P. Allostasis: a model of predictive regulation. Physiol Behav 106, 5–15 (2012). 10.1016/j.physbeh.2011.06.004 21684297

[56] Pezzulo G., Rigoli F. & Friston K. Active Inference, homeostatic regulation and adaptive behavioural control. Prog Neurobiol 134, 17–35 (2015). 10.1016/j.pneurobio.2015.09.001 26365173 PMC4779150

[57] Damasio A. R. Descartes’ Error. (Putnam, 1994).

[58] Tranel D., Bechara A., Damasio A. R. & Gazzaniga M. S. in The New Cognitive Neurosciences Vol. 2 1047–1061 (MIT Press, 2001).

[59] Dosenbach N. U. F., Raichle M. E. & Gordon E. M. The brain’s action-mode network. Nat Rev Neurosci 26, 158–168 (2025). 10.1038/s41583-024-00895-x 39743556

[60] Reiss S., Peterson R. A., Gursky D. M. & McNally R. J. Anxiety sensitivity, anxiety frequency and the prediction of fearfulness. Behav Res Ther 24, 1–8 (1986). 10.1016/0005-7967(86)90143-9 3947307

[61] Parvizi J. & Kastner S. Promises and limitations of human intracranial electroencephalography. Nat Neurosci 21, 474–483 (2018). 10.1038/s41593-018-0108-2 29507407 PMC6476542

[62] Liu S. & Parvizi J. Cognitive refractory state caused by spontaneous epileptic high-frequency oscillations in the human brain. Sci Transl Med 11 (2019). 10.1126/scitranslmed.aax7830 31619544

[63] Lusk Z. Combining Clinical Evaluations and Neuroscience Research in the Human Intracranial Electroencephalography Practice: 15-Year Cohort Study. J Cogn Neurosci, 1–18 (2025). 10.1162/jocn.a.53 40392101

[64] Groppe D. M. iELVis: An open source MATLAB toolbox for localizing and visualizing human intracranial electrode data. J Neurosci Methods 281, 40–48 (2017). 10.1016/j.jneumeth.2017.01.022 28192130

[65] Fischl B. FreeSurfer. NeuroImage 62, 774–781 (2012). 22248573 10.1016/j.neuroimage.2012.01.021 PMC3685476

[66] Jenkinson M., Bannister P., Brady M. & Smith S. Improved optimization for the robust and accurate linear registration and motion correction of brain images. Neuroimage. 17, 825–841. (2002). 12377157 10.1016/s1053-8119(02)91132-8

[67] Papademetris X. BioImage Suite: An integrated medical image analysis suite: An update. The insight journal 2006, 209 (2006). 25364771 PMC4213804

[68] Kurth F., Zilles K., Fox P. T., Laird A. R. & Eickhoff S. B. A link between the systems: functional differentiation and integration within the human insula revealed by meta-analysis. Brain Struct Funct 214, 519–534 (2010). 10.1007/s00429-010-0255-z 20512376 PMC4801482

[69] Amunts K., Mohlberg H., Bludau S. & Zilles K. Julich-Brain: A 3D probabilistic atlas of the human brain’s cytoarchitecture. Science 369, 988–992 (2020). 10.1126/science.abb4588 32732281

[70] Esterman M., Noonan S. K., Rosenberg M. & Degutis J. In the zone or zoning out? Tracking behavioral and neural fluctuations during sustained attention. Cereb Cortex 23, 2712–2723 (2013). 10.1093/cercor/bhs261 22941724

[71] Kucyi A. Electrophysiological dynamics of antagonistic brain networks reflect attentional fluctuations. Nat Commun 11, 325 (2020). 10.1038/s41467-019-14166-2 31949140 PMC6965628

[72] Lyu D., Stiger J. R., Lusk Z., Buch V. & Parvizi J. Mapping human thalamocortical connectivity with electrical stimulation and recording. Nat Neurosci (2025). 10.1038/s41593-025-02009-x PMC12664536 40664975

